# Effect of biomechanical properties on myopia: a study of new corneal biomechanical parameters

**DOI:** 10.1186/s12886-020-01729-x

**Published:** 2020-11-19

**Authors:** Fang Han, Mengdi Li, Pinghui Wei, Jiaonan Ma, Vishal Jhanji, Yan Wang

**Affiliations:** 1 Clinical College of Ophthalmology, Tianjin Medical University, No.4, Gansu Road, Heping District, Tianjin, 300020 China; 2 Department of Ophthalmology, The 1st People’s Hospital of Yunnan Province, Jinbi Rd 157#, Kunming, 650031 China; 3 Department of Ophthalmology, The Affiliated Hospital of Kunming Science and Technology University, Jinbi Rd 157#, Kunming, 650031 China; 4 Tianjin Eye Hospital, Tianjin Eye Institute, Tianjin Key Laboratory of Ophthalmology and Visual Science, Nankai University Affiliated Eye Hospital, Tianjin, China; 5 UPMC Eye Center, University of Pittsburgh School of Medicine, Pittsburgh, PA USA

**Keywords:** Corneal stress-strain index, Myopia, Corneal biomechanics

## Abstract

**Background:**

To assess the corneal stress-strain index (SSI), which is a marker for material stiffness and corneal biomechanical parameters, in myopic eyes.

**Methods:**

A total of 1054 myopic patients were included in this study. Corneal visualisation Scheimpflug technology was used to measure the SSI. Corneal biomechanics were assessed using the first and second applanation times (A1-and A2-times); maximum deflection amplitude (DefAmax); deflection area (HCDefArea); the highest concavity peak distance (HC-PD), time (HC-time), and deflection amplitude (HC-DefA); integrated radius (IR); whole eye movement (WEM); stiffness parameter (SP-A1;, biomechanically corrected intraocular pressure (BIOP); and Corvis biomechanical index (CBI). Scheimpflug tomography was used to obtain the mean keratometery (Km) and central corneal thickness (CCT). According to the spherical equivalent (SE) (low myopia: SE ≥ − 3.00D and high myopia: SE ≤ − 6.00D.), the suitable patients were divided into two groups.

**Results:**

The mean SSI value was 0.854 ± 0.004. The SSI had a positive correlation with A1-time ((*r* = 0.272), HC-time (*r* = 0.218), WEM (*r* = 0.288), SP-A1 (*r* = 0.316), CBI (*r* = 0.199), CCT (*r* = 0.125), bIOP (*r* = 0.230), and SE (*r* = 0.313) (all *p*-values<0.01). The SSI had a negative correlation with HCDefA (*r* = − 0.721), HCDefArea (*r* = − 0.665), HC-PD(*r* = − 0.597), IR (*r* = − 0.555), DefAmax (*r* = − 0.564), and Km (*r* = − 0.103) (all p-values<0.01). There were significant differences in SSI (t = 8.960, p<0.01) and IR (t = − 3.509, p<0.01) between the low and high myopia groups.

**Conclusions:**

In different grades of myopia, the SSI values were lower in eyes with higher SEs. It indicates that the mechanical strength of the cornea may be compromised in high myopia. The SSI was positively correlated with the spherical equivalent, and it may provide a new way to study the mechanism of myopia.

## Background

Quantification of corneal biomechanics has helped us to understand the changes in corneal shape and structure after refractive surgery [1, 2]. The corneal stiffness is a recently described index with clinical significance for the detection in patients who are at risk of ectasia development [3, 4]. Previous studies have shown that the biomechanical properties of the cornea are correlated with many factors such as central corneal thickness (CCT) and intraocular pressure (IOP) [5, 6]. By using finite element models of wide ranges of geometries of human eyeballs affected by different levels of intraocular pressure, Eliasy et al. [7] studied clinical data obtained from two large datasets of healthy participants. According to their algorithm, the study produced a material stiffness parameter-stress-strain index (SSI) as a new parameter does not show significant correlation with CCT and IOP.

However, the distribution characteristics and influencing factors of SSI in myopic patients have not been reported. In this study, we aimed to determine the normative values in myopic patients and the effect of SSI on myopia and to assess its possible correlation with other corneal biomechanical parameters.

## Methods

### Subjects

This was a retrospective clinical study of 1054 myopic participants (464 women and 590 men) who were scheduled to undergo corneal refractive surgery at Tianjin Eye Hospital, Tianjin Medical University between March 2019 and November 2019. Data from a single representative eye per participant (right eye) were used for the analysis.

According to the spherical equivalent (SE; low myopia: SE ≥ − 3.00 D and high myopia: SE ≤ − 6.00 D), suitable patients were divided into two groups. The study protocol followed the tenets of the Declaration of Helsinki and was approved by the ethics committee of Tianjin Eye Hospital. Written informed consent was obtained from all participants before enrolment. Inclusion criteria included: stable refraction for at least 2 years and the absence of ocular inflammation. Patients were asked to refrain from using soft contact lenses for at least 2 weeks and rigid contact lenses for at least 4 weeks. Exclusion criteria included: history of ophthalmic surgery, ocular trauma, keratoconus, glaucoma, diabetes, systemic connective tissue disease and abnormal immune function.

### Methods

All the participants underwent comprehensive eye examinations, including uncorrected visual acuity and best corrected visual acuity measurements, subjective refraction, non-contact tonometry, and slit lamp examinations. Corneal thicknesses and mean curvatures were obtained using Scheimpflug imaging (Pentacam, Oculus, Germany). Corneal biomechanical parameters were obtained using the corneal visualisation Scheimpflug technology (Corvis ST) analyser (Oculus, Germany).

### Corvis ST and SSI

**Fig. 1:**
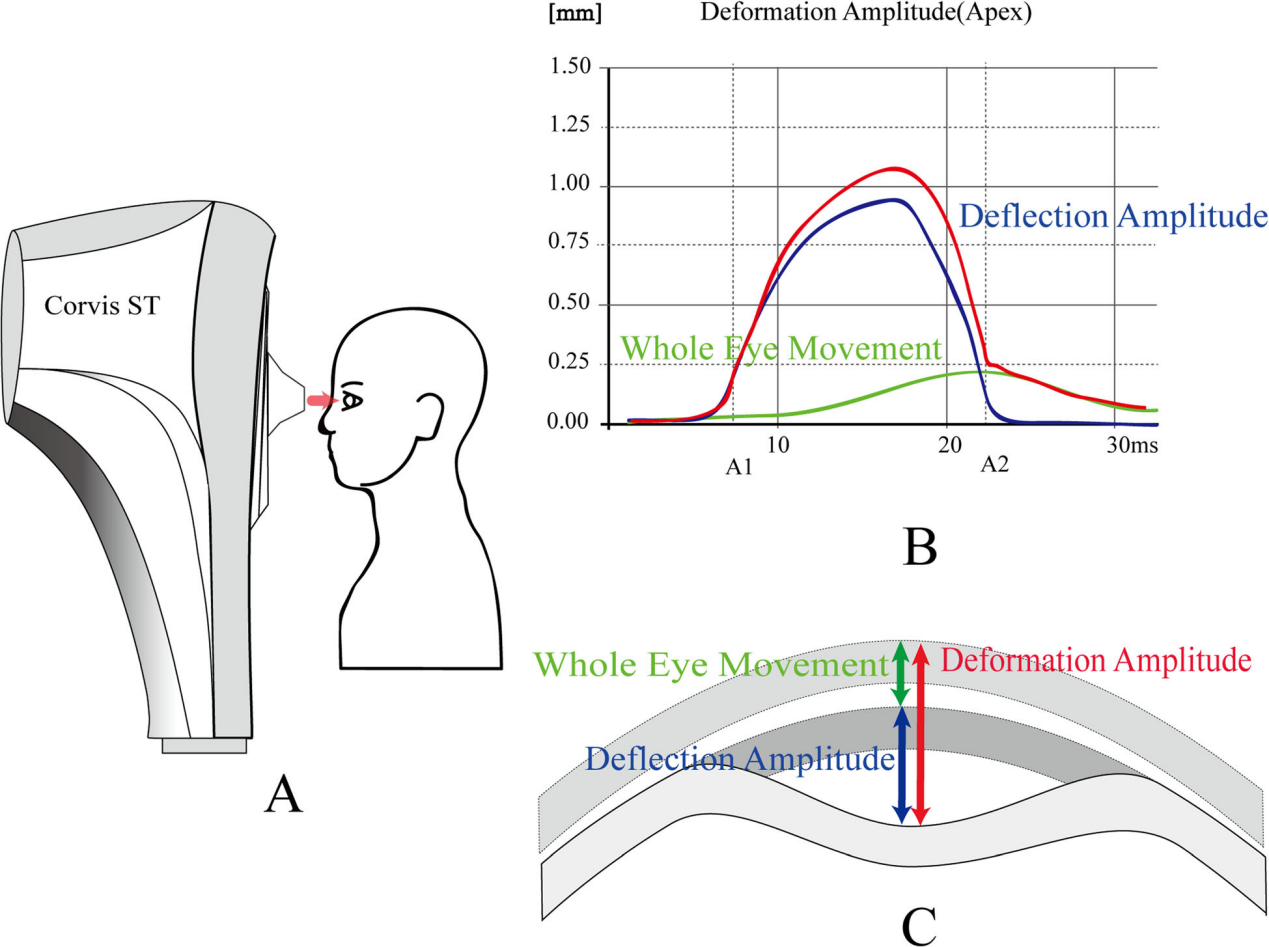
Schematic view of the Corvis ST test and cornea deformations. The air puff impinges (pink line) on the corneal surface, the cornea becomes concave and whole eye motion (green line) is simultaneously initiated in the backward direction. Deformation amplitude: deformation of the corneal apex (red line). Deflection amplitude (mm): displacement of corneal apex y after eye motion in removed (blue lines). Corvis ST: corneal visualisation Scheimpflug technology

The CorvisST (software version 1.6r2015) is a non-contact and visual dynamic intraocular pressure analyzer, which integrates ultra-high-speed Seheimpflug technology into a non-contact intraocular pressure measuring instrument to study the whole dynamic process of corneal deformation under external force. The parameters of the deformation process are recorded, and the biomechanical properties of the cornea are analyzed (Fig. 1). The new software used in this study measures new corneal biomechanical index SSI, included in the dynamic corneal response (DCR) parameters, which consist of first applanation (A1) parameters (A1-time, A1-length and A1-velocity), second applanation (A2) parameters (A2-time, A2-length and A2-velocity), highest concavity (HC) parameters (HC-time, HC-radius, HC deformation amplitude (DA), HC peak distance (PD), maximum deflection amplitude (DefAmax), deflection area (HCDefArea), corneal stiffness parameter (SP-A1), biomechanically corrected intraocular pressure (bIOP), and Corvis biomechanical index (CBI) as well.

The value of SSI was estimated by an in-built software using the least squares method, according to numerical modelling using CCT, bIOP, and SP-HC as input and output parameters [7]. It fits the data with the previous numerical analysis results by the regression equation.

$$ \mathrm{SSI}=\mathrm{f}\ \Big(\mathrm{a}1+\mathrm{a}2\mathrm{C}1+\mathrm{a}3\mathrm{C}2+\mathrm{a}4{\mathrm{C}}_1^2+\mathrm{a}5\mathrm{C}1\mathrm{C}2+\mathrm{a}6{\mathrm{C}}_2^2+\mathrm{a}7{\mathrm{C}}_1^3+\mathrm{a}8{\mathrm{C}}_1^2\mathrm{C}2+\mathrm{a}9\mathrm{C}1{\mathrm{C}}_2^2+{\mathrm{C}}_2^3+\ln \left(\mathrm{SP}-\mathrm{HC}\right) $$

where C1 = CCT/545 and C2 = bIOP/20. ln (SP-HC) the natural logarithm of the SP at HC, and a1-a9 constants are determined by fitting the equation to the numerical input and output values. The ssi was considered as 1.0 for the average experimental behaviour obtained for corneal tissue with age = 50 years [8]. Higher values of SSI are indicative of higher tissue stiffness and vice versa.

### Statistical analyses

The statistical analyses were performed using SPSS version 26.0 software (IBM, Corp, Armonk, NY, USA). Descriptive statistical results included means, standard deviations, and minimum and maximum values of parameters. The 95% confidence interval (CI) of the overall mean of the parameters was calculated. Normality of all data samples was checked using the Kolmogorov-Smirnov test. Pearson bivariate correlation statistical analysis was used to obtain the linear fit of the correlation among variables. Stepwise multivariate linear regression analysis was applied to assess the correlation between SSI and other corneal properties; *p*<0.05 was considered to be statistically significant.

## Results

### Baseline characteristics

Data were collected from 1054 patients. The right eyes were used for the analysis. The characteristics of the participants are summarised in Table 1. The mean age and SE of the participants were 23.9 ± 5.96 years and − 5.03 ± 2.03D, respectively.

**Table 1:** Characteristics of the participants included in the study

Parameters	Mean ± SD	Range
Age (years)	23.9 ± 5.96	17–45
MRSE (D)	−5.36 ± 2.07	− 0.5- -14.25
Spherical (D)	−5.03 ± 2.03	−0.5- -13.50
Cylinder (D)	−0.66 ± 0.48	0- -1.75
CCT (microns)	553.4 ± 29.9	482–654
Km (D)	43.14 ± 1.35	39.0–46.75
IOPnct (mmHg)	16.39 ± 2.37	9.5–27.5

Values are presented as means (standard deviations) or as ranges

*CCT* Central corneal thickness, *Km* Mean keratometry, *MRSE* Manifest refraction spherical equivalent, *IOPnct* Intraocular pressure with non-contact tonometry, *SD* Standard deviation

### Biomechanical parameters

The mean values of DCR parameters in eyes with corresponding standard deviations and 95% CIs are shown in Table 2.

**Table 2:** Distribution of normative values of Corvis ST parameters

Parameters	Mean ± SD	95% CI
A1-time (ms)	7.208 ± 0.289	7.194,7.223
A2-time (ms)	21.968 ± 0.373	21.949,21.987
HC-time (ms)	17.032 ± 0.401	17.011,17.052
A1DefA (mm)	0.097 ± 0.006	0.096,0.097
A2DefA (mm)	0.109 ± 0.011	0.108,0.109
HCDefA (mm)	0.941 ± 0.096	0.936,0.946
HCDefArea (mm^2^)	3.521 ± 0.501	3.495,3.547
DefAmax (mm)	0.953 ± 0.095	0.948,0.957
DAmax (mm)	4.349 ± 0.424	4.328,4.372
PD (mm)	5.181 ± 0.251	5.169,5.194
WEM (mm)	0.201 ± 0.065	0.258,0.265
SP-A1	107.866 ± 14.927	107.091,108.640
ARTh	581.715 ± 112.10	575.918,587.512
IR	7.902 ± 0.977	7.852,7.951
CBI	0.221 ± 0.187	0.212,0.230
SSI	0.854 ± 0.133	0.847,0.860
CCT (μm)	554 ± 32.75	576,587
bIOP (mmHg)	16 ± 2.06	15.9,16.1

Values are presented as means (standard deviations) with 95% confidence intervals

*Corvis ST* Corneal visualisation Scheimpflug technology, *SD* Standard deviation, *CI* Confidence interval, *A1-and A2-times* Time reaching the first and second applanation, *HC-time* Highest concavity-time, *A1 and A2DefA* Displacement of corneal apex at the first or second applanation or at the moment of highest concavity after whole eye motion is removed, *HCDefA* Amplitude at the highest concavity, *HCDefArea* Deflection area at the highest concavity, *DefAmax* Maximum deflection amplitude, *DAmax* Maximum deformation amplitude, *PD* Peak distance, *WEM* Whole eye movement, *SP-A1* Stiffness parameter, *IR* Integrated radius, *ARTh* Ambrosio relational thickness horizontal, *CBI* Corvis biomechanical index, *SSI* Stress-strain index, *CCT* Central corneal thickness, *bIOP* Biomechanically corrected intraocular pressure

### Correlations of SSI with other biomechanical parameters

#### Correlation between baseline characteristics and SSI

**Fig. 2:**
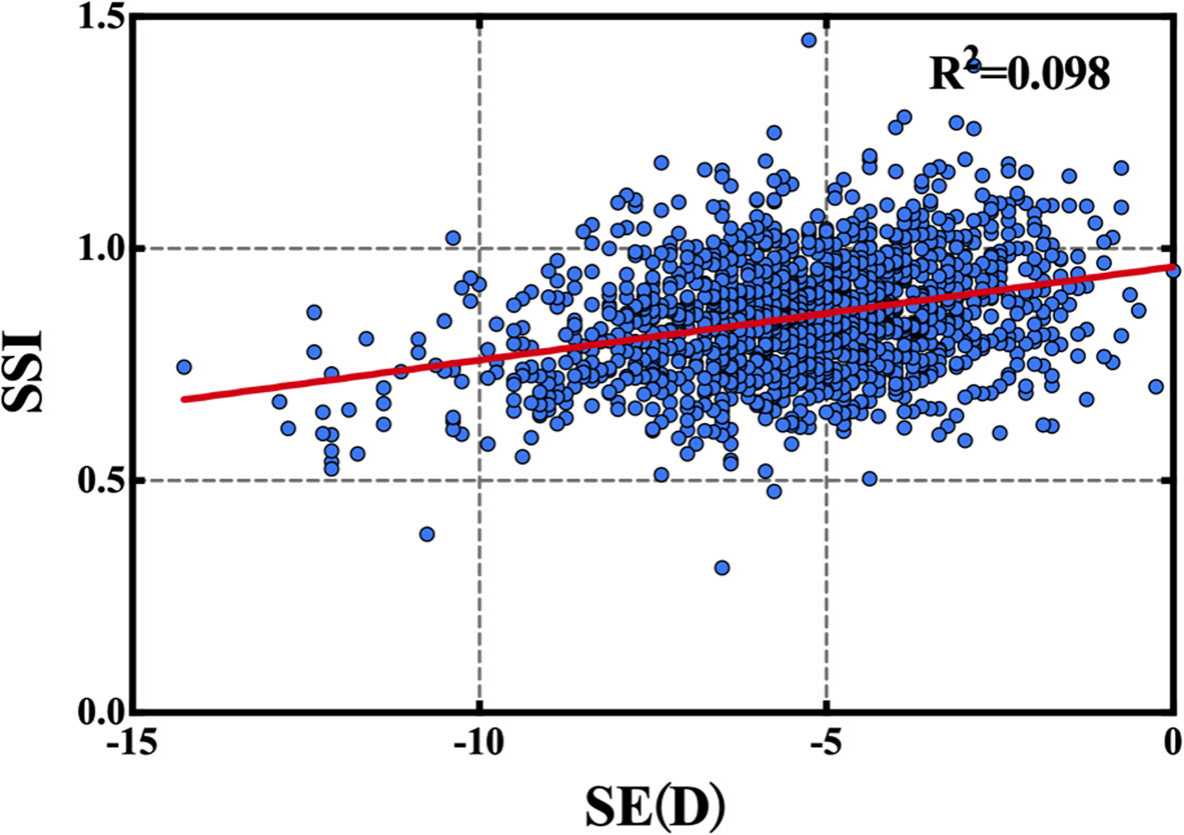
Scatter plots showing significant correlation between SSI and spherical SE (*r* = 0.313, *p*<0.01). SSI: stress-strain index; SE: spherical equivalent

No statistically significant correlations were observed between SSI and sex (*p*>0.05). The SSI was negatively correlated with mean keratometry (Km) (*r* = − 0.103, *p*<0.01) and positively correlated with SE (*r* = 0.313, *p*<0.01) (Fig. 2), CCT (*r* = 0.125, *p*<0.01), age (*r* = 0.198, *p*<0.01), and bIOP (*r* = 0.23, *p*<0.01) (Table 3).

**Table 3:** Correlations between SSI and characteristics of the participants

Parameters	SSI	
	r	*p*
Age (years)	0.198	<0.01
MRSE (D)	0.313	<0.01
CCT (microns)	0.125	<0.01
Km (D)	−0.103	<0.01
bIOP (mmHg)	0.230	<0.01

Statistical significance has been defined as *p* < 0.05

*SSI* Stress-strain index, *MRSE* Manifest refraction spherical equivalent, *CCT* Central corneal thickness, *Km* Mean keratometry, *BIOP* Biomechanically corrected intraocular pressure

#### Comparison of parameters in low and high myopia

**Fig. 3:**
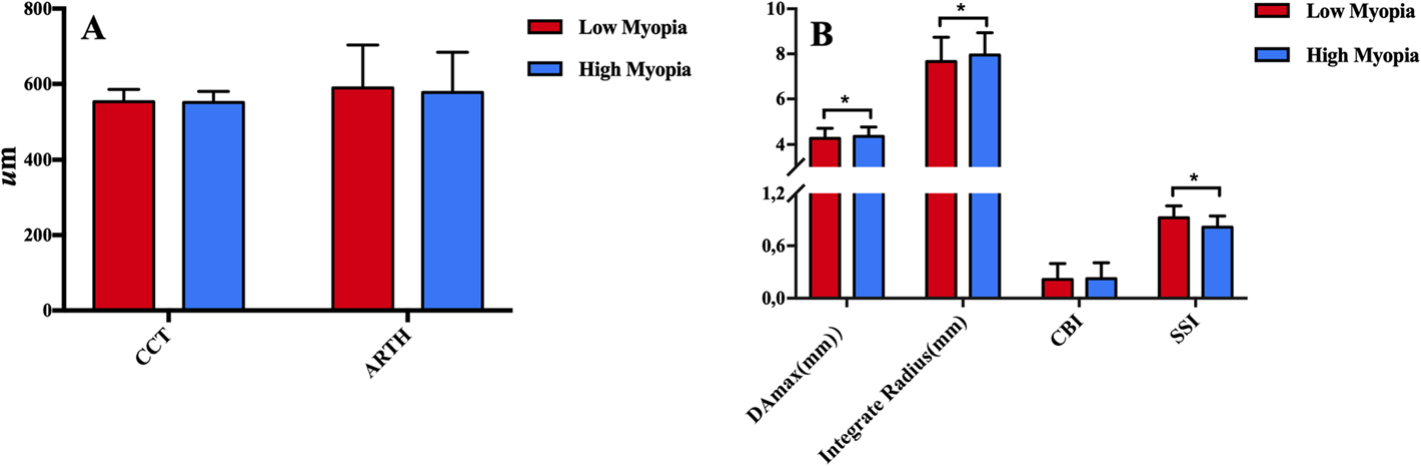
Histogram comparison of parameters in low and high myopia. The x axis represents the parameters for comparison between low and high myopia. The y axis represents the numerical value of the paramete. CCT: central corneal thickness; ARTh: ambrosio relation thickness horizontal DAmax: maximum deformation amplitude; CBI: Corvis biomechanical index; SSI: stress-strain index

There were no significant differences in age, CCT, BIOP, SP, ambrosio relational thickness horizontal (ARTh), and CBI between the low and high myopia groups. There was a significant difference in SSI (t = 8.960, *p*<0.01) and integrated radius (IR)(t = − 3.509, *p*<0.01) (Fig. 3, Table 4).

**Table 4:** Comparison of the parameters in low and high myopia

Group	Number	Age (years)	CCT (microns)	bIOP	SP-A1	DAmax (mm)	IR (mm)	ARTh	CBI	SSI
Low myopia	161	23.2 ± 6.12	553 ± 32.9	16 ± 2.07	108.520 ± 15.699	4.272 ± 0.448	7.663 ± 1.071	589.402 ± 114.500	0.214 ± 0.184	0.920 ± 0.138
High myopia	506	24.0 ± 5.56	551 ± 29.3	16 ± 1.98	107.787 ± 14.884	4.360 ± 0.413	7.953 ± 0.991	577.718 ± 106.814	0.224 ± 0.183	0.813 ± 0.129
t		−1.616	0.770	−1.354	0.538	−2.328	−3.509	1.190	−0.609	8.960
*p*		0.107	0.441	0.176	0.590	0.020	<0.01	0.234	0.543	<0.01

Statistical significance has been defined as *p* < 0.05

*CCT* Central corneal thickness, *bIOP* Biomechanically corrected intraocular pressure, *SP-A1* Stiffness parameter, *DAmax* Maximum deformation amplitude, *IR* Integrated radius, *ARTh* Ambrosio relational thickness horizontal, *CBI* Corvis biomechanical index, *SSI* Stress-strain index

#### Regression analysis of SSI and baseline characteristics

Multiple linear stepwise regression analysis was performed with SSI as the dependent variable and SE, Km, CCT, bIOP, and age as independent variables (Table 5). The following regression equation was obtained:

$$ \mathrm{SSI}=0.768+0.021\mathrm{SE}+0.02\mathrm{bIOP}+0.006\mathrm{AGE}-0.013\mathrm{KM}+0.001\mathrm{CCT} $$

**Table 5:** Multiple linear stepwise regression analysis with SSI as the dependent variable

	β	t	*P*	95% CI
Constant	0.768	6.429	<0.01	0.534,1.002
MRSE (D)	0.021	14.114	<0.01	0.018,0.024
bIOP	0.020	12.752	<0.01	0.017,0.023
Age (year)	0.006	11.352	<0.01	0.005,0.007
Km (D)	-0.013	−5.680	<0.01	−0.018, − 0.009
CCT (μm)	0.001	5.558	<0.01	0.000,0.001

Values are presented 95% confidence intervals, and statistical significance has been defined as *p* < 0.05

*CI* Confidence interval, *MRSE* Manifest refraction spherical equivalent, *bIOP* Biomechanically corrected intraocular pressure, *Km* Mean keratometry, *CCT* Central corneal thickness, *SSI* Stress-strain index

#### Correlation between corneal biomechanical parameters and SSI

**Fig. 4:**
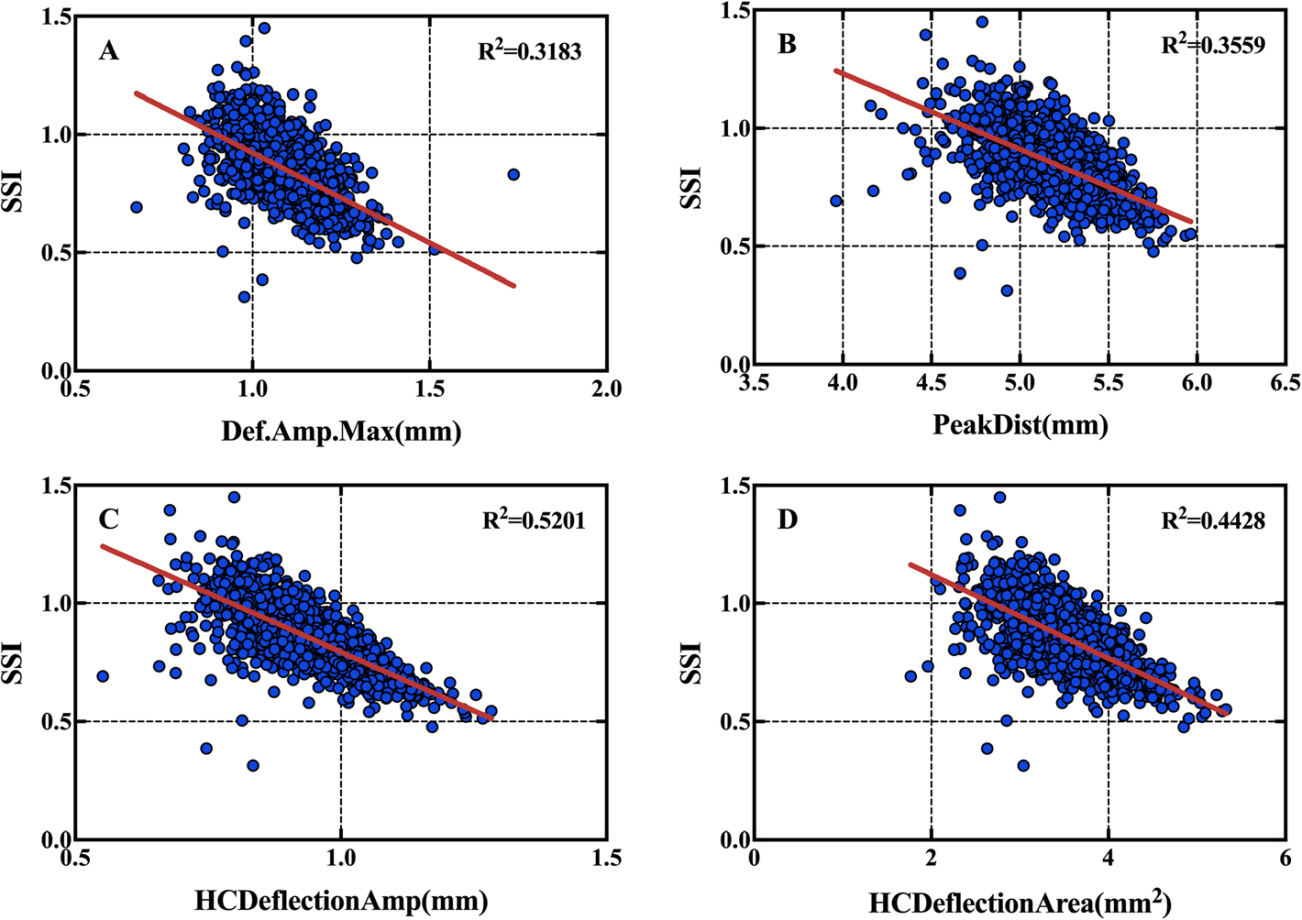
Scatter plots showing correlations of SSI with DefAmax (*r* = − 0.564, *p*<0.01), Peakdist (*r* = − 0.597, *p*<0.01), HCDefA (*r* = − 0.721, *p*<0.01), HCDefArea (*r* = − 0.665, *p*<0.01). SSI: stress-strain index; DefAmax: maximum deflection amplitude; PD: Peak distance; HCDefA: highest concavity deflection amplitude; HCDefArea: highest concavity deflection area

A1-time, HC-time, A2DefA, WEM, SP-A1, ARTh, CBI, IR, and bIOP were weak positively correlated with SSI. DAmax, A2-time, and A2DefA were weakly negatively correlated with SSI. HCDefA, HCDefArea, PD, IR, and DefAmax were strongly negatively correlated with SSI. No significant correlation was found between A1DefA and SSI (Table 6, Fig. 4).

**Table 6:** Correlations between SSI and Corvis ST parameters

Parameters	SSI	
	R	*p*
A1-time (ms)	0.272	<0.01
A2-time (ms)	−0.323	<0.01
HC-time (ms)	0.218	<0.01
A1DefA (mm)	−0.007	0.798
A2DefA (mm)	0.081	<0.01
HCDefA (mm)	−0.721	<0.01
HCDefArea (mm^2^)	−0.665	<0.01
DefAmax (mm)	−0.564	<0.01
DAmax (mm)	−0.388	<0.01
PD (mm)	−0.597	<0.01
WEM (mm)	0.288	<0.01
SP-A1	0.316	<0.01
ARTh	0.113	<0.01
IR	−0.555	<0.01
CBI	0.199	<0.01
bIOP	0.230	<0.01

Statistical significance has been defined as *p* < 0.05

*SSI* Stress-strain index, *Corvis ST* Corneal visualiation Scheimpflug technology, *A1-and A2-times* Time reaching the first and second applanation, *HC-time* Highest concavity-time, *A1 and A2DefA* Displacement of corneal apex at the first or second applanation or at the moment of highest concavity after whole eye motion is removed, *HCDefA* Amplitude at the highest concavity, *HCDefArea* Deflection area at the highest concavity, *DefAmax* Maximum deflection amplitude, *DAmax* Maximum deformation amplitude, *PD* Peak distance, *WEM* Whole eye movement, *SP-A1* Stiffness parameter, *IR* Integrated radius, *ARTh* Ambrosio relational thickness horizontal, *CBI* Corvis biomechanical index, *bIOP* Biomechanically corrected intraocular pressure

## Discussion

Myopia is a public health problem [9] with a high prevalence, especially in the Far East [10]. The onset and progression of myopia has been associated with genetic and environmental factors [11, 12]. Previous studies have noted that myopia is correlated with an increase in corneal curvature and a decrease in corneal thickness [13]. Animal studies have shown a change in the length of the eye and shape of the anterior cornea during the process of myopia modelling [14, 15]. It is also known that high myopes have lower corneal hysteresis than emmetropes [16]. However, it is difficult to detect the ocular biomechanical properties in vivo [17]. The Corvis ST provides information on corneal deformation parameters by visualising the dynamic reaction of the cornea to a single puff of air [18].

In our study, one of the new Corvis ST parameters—SSI, was evaluated in myopic eyes. We demonstrated that the SSI was positively correlated with SE (*r* = 0.313, *p*<0.01) (Fig. 1). When comparing eyes with low and high myopia, there were no significant differences in CCT, bIOP, SP, ARTh, and CBI, although there was a significant difference in SSI(t = 8.960, *p*<0.01) and IR(t = − 3.509, *p*<0.01 (Fig. 2, Table 4) values. The results showed that the SSI of high myopia was lower than that of low myopia, suggesting that the biomechanical properties of the cornea changed and corneal hardness decreased with an increase in the SE.

Inmaculada Bueno-Gimeno et al. used ocular response analyser and suggested that corneal biomechanical properties appear to be compromised in myopia from an early age, especially in high myopia [19]. Another study showed a weak although significant correlation between corneal hysteresis (CH) and refractive error, with CH being lower in both moderate and high myopia than in emmetropia and low myopia [20]. Wu et al. [21] reported a difference in corneal biomechanical properties between 835 low myopic eyes and 1027 high myopic eyes. Low CH and corneal resistance factor and high cornea-compensated and Goldmann-correlated IOPs were suggested to be associated with high myopia. However, the correlation of the biomechanics of myopia is controversial. Some studies reported no significant correlation between myopia and CH [22, 23]. The results of our study showed a strong negative correlation of SSI with HCDefA(*r* = − 0.721, *p*<0.01), HCDefArea(*r* = − 0.665, *p*<0.01), PD(*r* = − 0.597, *p*<0.01), IR(*r* = − 0.555, *p*<0.01), and DefAmax(*r* = − 0.564, *p*<0.01) (Table 6, Fig. 3). Wang et al. [24] found that eyes with high myopia had a larger corneal DA than eyes with mild-to-moderate myopia, and A2-time and HC-radius were positively correlated with SE. Eyes with high myopia also showed longer DA and smaller HC-radius. Similar results were reported by Miaohe et al. [5]. These findings are consistent with our results.

Previous studies have shown that the biomechanical properties of the cornea are correlated with CCT. Eyes with thick CCT exhibited strong corneal resistance to external force and are less prone to deformation [25]. Higher intraocular pressure may mask abnormal corneal biomechanical properties, resulting in apparently normal HCDA measurements [26]. The introduction of SSI resolved this issue because it estimates the material stiffness [27]. Eliasy et al. [7] used a numerical model in the study of SSI parameter, which not only covered a wide range of changes in IOP, CCT, geometry and material parameters, but also covered even slightly extended beyond the scope reported in clinical studies. Through the consideration of a Corvis parameter—SP-HC, it is more strongly correlated with corneal stiffness than IOP. As a new index independent of IOP and corneal geometry, SSI could specifically detect high-risk or susceptible patients with ectasia after refractive surgery, remind physicians of the possible risks caused by the decrease of corneal biomechanical properties and could aid in surgery planning.

In this study, we observed a weak correlation among BIOP (*r* = 0.23, *p*<0.01), CCT (*r* = 0.125, *p*<0.01), and SSI (Table 3). It indicated that despite correction, the effect of corneal biomechanics cannot be completely independent of IOP and thickness, which is consistent with our clinical experience and previous studies. Effects of IOP and corneal biomechanics on eye behaviour are difficult to separate; IOP also affects the immediate corneal stiffness. It is generally believed that sex has no significant effect on corneal biomechanics [28]. Our study also suggested that the corneal biomechanical properties of myopia may have nothing to do with sex. Correlation between stress-strain behaviour and age was reported [8, 29], although this study found that there was no strong significant correlation between age (*r* = 0.198, *p*<0.01) and SSI, which may be correlated with the concentration of the individual age included.

It is noteworthy that the mean CCT value measured by Corvis ST (553 ± 29.96 um) was slightly lower than that measured by Pentacam corneal topography (554 ± 31.04 um) (t = 4.970 *p*<0.01). However, it has been shown that Corvis-ST CCT measurements have good repeatability [30].

The main limitation of the current study is lack of eye axis parameters and a control group with emmetropia, despite a large sample size with myopic participants, which will be improved and supplemented in future studies.

## Conclusions

In conclusion, our study showed that there was a positive correlation between SSI and SE. It may provide a new way to study the mechanism of myopia. In different grades of myopia, the SSI values were lower in eyes with higher SE. This indicates that the mechanical strength of the cornea may by compromised in high myopia. Future studies can corroborate the findings of our study. A longitudinal study in progressive and stable myopic participants is warranted.

## Data Availability

The datasets during and/or analysed during the current study are available from the corresponding author on reasonable request.

## Abbreviations

A1-time: The first and applanation time
A2-time: The second applanation time
ARTh: Ambrosio relational thickness horizontal
bIOP: Biomechanically corrected intraocular pressure
CBI: Corvis biomechanical index
CH: Corneal hysteresis
Corvis ST: Corneal visualisation Scheimpflug technology
CCT: Central corneal thickness
CI: Confidence interval
DA: Deformation amplitude
DCR: Dynamic corneal response
DefAmax: Maximum deflection amplitude
HCDefArea: Deflection area
IR: Integrated radius
Km: Mean keratometery
IOP: Intraocular pressure
PD: Peak distance
SE: Spherical equivalent
SP-A1: Stiffness parameter
SP-HC: Stiffness parameter at highest concavity
SSI: Stress-strain index
WEM: Whole eye movement
